# FastSAM-Based Automated Segmentation and Data Extraction for Pore Structures of Foamed Concrete

**DOI:** 10.3390/ma19153215

**Published:** 2026-07-28

**Authors:** Luchang Xiong, Siyu Du, Zhijun Wan, Xuan Cui, Bingrui Chen, Yu Huang, Zhonghua Sun

**Affiliations:** 1 School of Safety Engineering, China University of Mining and Technology, Xuzhou 221116, China; 2 School of Mines, China University of Mining and Technology, Xuzhou 221116, China; zhjwan@cumt.edu.cn (Z.W.); xcui@cumt.edu.cn (X.C.); cbrtb@cumt.edu.cn (B.C.); 3 SPIC (Zunyi) Industrial Development Co., Ltd., Zunyi 564300, China; huangyu03@spic.com.cn (Y.H.); sunzhonghua@spic.com.cn (Z.S.)

**Keywords:** low-carbon concrete, foamed concrete, FastSAM, pore structure, image segmentation, Douglas–Peucker algorithm

## Abstract

Reliable pore-structure recognition and descriptor extraction from foamed concrete micrographs remain challenging because pore walls are blurred, adjacent pores are often connected, and image statistics vary with observation scale. This study presents a FastSAM-DP workflow for automatic pore segmentation and pore-structure assessment. FastSAM generates pore instance masks, the Douglas–Peucker (DP) algorithm regularizes and simplifies contour geometry, and the workflow extracts the pore-size coefficient of variation (CV), circularity (Ci), number density (N), uniformity index (UI), large-pore area fraction (FL), and an image-derived composite pore-structure descriptor (PSQI). Configuration was selected using development data and assessed by source-filename-group held-out internal evaluation. In the complete 25-group/100-file held-out set, FastSAM-DP achieved an instance F1 of 0.732 (95% CI, 0.696–0.767), compared with 0.050 for fixed Otsu–Watershed; PSQI agreement was r = 0.828 (95% CI, 0.665–0.917), with MAE = 7.94 and RMSE = 11.10. In the post-audit 14-group/56-file non-overlap sensitivity subset, instance F1 was 0.752 (95% CI, 0.713–0.791) and the PSQI agreement was r = 0.838 (95% CI, 0.585–0.942), with MAE = 7.41 and RMSE = 10.50. Multiscale analysis included 130 images in 107 conservative image-field partitions at 15×, 20× and 40×, treated as independent descriptive strata. The workflow is therefore intended for batch pore-structure screening and same-magnification comparison within the present internal dataset.

## 1. Introduction

Foamed concrete is a lightweight cementitious material used in thermal insulation, goaf backfilling, tunnel grouting and lightweight components because of its low density, thermal insulation capacity and pumpable filling behavior [1,2,3,4]. Unlike dense cementitious materials, its macroscopic performance is strongly governed by the mesoscopic pore structure. Pore-size distribution, pore-shape regularity, pore-number density, spatial uniformity and the fraction of large defects jointly affect solid-skeleton continuity, local stress concentration and heat-transfer pathways [5,6,7]. Foam stability and slurry rheology further influence bubble generation, coalescence and pore retention [8,9]. Interfacial regulation and hydrophobization strategies have also been used to improve foam stability and hardened-structure continuity [10,11]. Changes in porosity and pore size can alter compressive strength, splitting strength, permeability and structural stability in porous concrete [12,13,14]. Pore-structure characterization should therefore go beyond representative images or a single porosity value. It requires repeatable conversion of micrographs into comparable quantitative descriptors. Such automated characterization is needed to explain how foaming agents, stabilizers and the water-to-solid ratio affect foamed concrete and to support mix optimization.

Micrograph analysis provides direct evidence for pore-structure studies of foamed concrete, but automatic pore recognition remains a limiting step in batch analysis. Conventional threshold segmentation, morphological processing and watershed algorithms rely mainly on grayscale contrast and manually selected parameters. They are therefore prone to under-segmentation or over-segmentation under uneven illumination, pore-wall shadows, connected pores and matrix-texture interference [15]. In recent years, U-Net, Mask R-CNN and related models have been applied to particles, pores and SEM microstructures in cement-based materials [16,17,18], demonstrating the value of deep learning for complex microstructure recognition. However, fully supervised models usually require extensive pixel-level or instance-level annotation. Their performance may also degrade when magnification, mix composition or imaging conditions change. For materials experiments with frequently changing sample batches, annotation burden and transfer uncertainty remain practical barriers [19,20,21].

Vision foundation models provide a route for reducing annotation dependence and improving cross-sample adaptability. The Segment Anything Model and its lightweight variant FastSAM can generate candidate instance masks without retraining for every image batch [22,23]. Recent image-based studies of cementitious materials have also incorporated SEM pore analysis, microstructure image enhancement and image-driven performance prediction into automated workflows, indicating a shift from qualitative observation to computable characterization [24,25,26]. Nevertheless, masks produced by foundation models are not automatically equivalent to material parameters. Pore boundaries in micrographs often contain pixel stair-steps, local burrs, blurred walls and connected contours. Without geometric correction, boundary-sensitive descriptors such as perimeter, circularity and large-pore fraction can be systematically amplified or weakened. Pore segmentation workflows therefore require contour correction designed for subsequent geometric calculation, not only visually plausible masks.

At the materials-assessment level, machine learning has been used to predict strength, permeability and composition–performance relations in foamed concrete and blended concretes [27,28,29,30]. Without interpretable pore-structure descriptors, however, these relations remain difficult to link back to material structure. A single pore size or porosity value cannot adequately describe pore quality in foamed concrete. Air-void characterization in foamed concrete and concrete air-void system evaluation usually require simultaneous consideration of air content, pore-size distribution, pore-shape features, spatial spacing and large-pore fraction [6,7,31]. Studies of fresh foam-concrete collapse and reviews of foamed-concrete technology further show that pore-structure quality is coupled with fresh foam stability, defect-pore formation and strength development after hardening [32,33]. On this basis, this study integrates CV, Ci, N, UI and FL into an integrated PSQI value for assessing pore-structure trends and screening mixes within the same analysis domain. For reproducibility, a single AHP-Entropy transform was fitted using development reference annotations and frozen before held-out evaluation and mixture-image analysis. Here, the PSQI is used as an image-scale auxiliary descriptor of pore structure, not as a substitute for strength, thermal conductivity or durability testing.

This study develops a FastSAM-DP workflow for automatic pore segmentation, parameter extraction and PSQI assessment of foamed concrete. The workflow uses FastSAM for candidate pore recognition, applies the Douglas–Peucker algorithm for contour regularization, and calculates CV, Ci, N, UI, FL, and PSQI. The study addresses three questions. First, segmentation performance is evaluated using development-only configuration selection and source-filename-group held-out internal evaluation; Otsu–Watershed is used as a quantitative classical baseline, whereas SAM is retained as a representative qualitative comparison. Second, pore-structure descriptors and the PSQI are calculated from both manual annotations and FastSAM-DP outputs to evaluate how automatic extraction reproduces reference annotations and where errors arise. Third, the HP, SF and WS mix series are analyzed at 15×, 20× and 40× observation magnifications to examine the scale sensitivity of the pore-structure parameters and PSQI. The resulting workflow is intended for bounded same-magnification mix screening and batch image assessment.

## 2. Materials and Methods

### 2.1. Image Data and Sample Grouping

Two types of micrograph data were used in this study. The algorithm-validation collection contained 473 annotated 640 × 640 JPG exports associated with 125 source-filename groups and calibrated records at 10×, 12×, 15×, 20× and 40×. A source-filename group was defined as all validation exports sharing the filename that precedes the ‘.rf.<hash>’ suffix. Development data comprised 373 files from 100 groups, whereas 100 files from 25 groups were reserved for source-filename-group held-out internal evaluation. A conservative image-field partition was defined as a connected set of raw image records judged identical, overlapping or ambiguous on the basis of filename provenance and geometric audit evidence. Post-audit overlap screening mapped validation groups to these partitions and excluded held-out groups whose partitions intersected development partitions, yielding a 14-group/56-file non-overlap sensitivity subset. The mix-analysis collection comprised 130 retained BMP images from the HP, SF and WS series at 15×, 20× and 40×, forming 107 conservative image-field partitions after two scale-conflicting records were excluded. No validation export and mix-analysis image was byte-identical by SHA-256; however, source-name tracing linked 108 validation groups (432 exports) to 108 of the 130 mix-analysis images. The collections therefore differ in file representation and analytical purpose but partly share underlying image provenance. The mix-analysis collection is treated only as descriptive and not as an independent validation cohort. The archive does not establish specimen-level, batch-level or cross-device independence, so the results are interpreted as internal methodological validation rather than independent external validation.

HP, SF and WS correspond to changes in the H_2_O_2_ chemical foaming agent dosage, calcium stearate stabilizer dosage and water-to-solid ratio (W/S), respectively. The matrix material used in this study is consistent with that used in the authors’ previous related studies [34,35]. Previous studies have shown that foaming agent, dry density, filler type and slurry composition can substantially affect pore retention and mechanical behavior in foamed concrete [36,37,38]. All three sample series were characterized using 15×, 20× and 40× micrographs. In the HP series, Binder: SF was fixed at 99:1 and W/S at 0.50. In the SF series, H_2_O_2_ was fixed at 5% of the total liquid mass, equivalent to 2.5% of the solid mass, and W/S at 0.50. In the WS series, Binder: SF was fixed at 99:1 and H_2_O_2_ at 5% of the total liquid mass. During data cleaning, two scale-conflicting SF01 files were excluded, and filename suffixes such as heavy, light, blue, green, rep and -1 were treated as archive descriptors rather than additional dose groups. Sample grouping and the numbers of valid images are listed in Table 1.

### 2.2. Overall Workflow for FastSAM-DP Automatic Pore Segmentation, Parameter Extraction and PSQI Assessment

**Figure 1:**
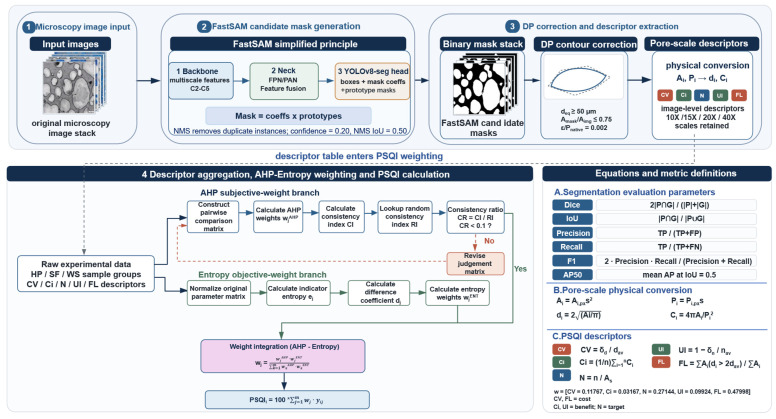
FastSAM-DP workflow for pore segmentation, parameter extraction and PSQI assessment.

The overall FastSAM-DP workflow for automatic pore segmentation, parameter extraction and PSQI assessment is shown in Figure 1. The workflow contains four modules: micrograph input and candidate-mask generation, Douglas–Peucker (DP) contour correction, pore-level physical-parameter and image-level descriptor calculation, and reliability assessment for segmentation and PSQI consistency. This structure is designed to convert visual segmentation outputs into pore-structure indicators for materials analysis, rather than stopping at mask visualization.

The automatic segmentation stage used a pretrained FastSAM model without additional training. Micrographs were resized consistently and input into the YOLOv8-seg framework. The model generated candidate instance masks through the backbone, feature-fusion layers and segmentation head. For FastSAM-s inference, micrographs were letterboxed to imgsz = 640, and masks were inverse-mapped to native geometry before physical filtering; development-only selection locked confidence = 0.20 and NMS IoU = 0.50 [22,23].

After inverse mapping to native coordinates, an equivalent diameter of 50 µm was used as the operational minimum analysis cutoff for both reference and predicted instances before instance matching, segmentation-metric calculation and descriptor calculation. Predictions were additionally required to have confidence ≥ 0.20 and an image-area ratio ≤ 0.75; these two filters were not applied to reference annotations. Reported segmentation and descriptor performance therefore pertains to pores within the defined analysis range of an equivalent diameter ≥ 50 µm. The DP algorithm was applied to each retained contour rather than activated by a pixel-perimeter threshold. The approximation tolerance was set to epsilon = 0.002 × native perimeter. This step was mainly used to reduce the influence of pixel stair-steps and local burrs on perimeter, circularity and large-pore fraction [39]; it did not split or merge pore instances.

After contour correction, pixel quantities were converted into physical quantities. For the ith pore, *A_i_*_,px_ denotes the pixel area, *P_i_*_,px_ denotes the pixel perimeter and *s* denotes the scale conversion factor. The actual area, actual perimeter, equivalent diameter and circularity were calculated using Equations (1)–(4). The audited native scale factors were 4.386, 3.663, 2.941, 2.146 and 1.081 µm px−1 for 10×, 12×, 15×, 20× and 40× records, respectively. Physical quantities were calculated only after inverse mapping to native coordinates.

(1) $$A_{i}=A_{i,\mathrm{px}}\;s^{2}$$

(2) $$P_{i}=P_{i,\mathrm{px}}\;s$$

(3) $$d_{i}=2\sqrt{\left(\frac{A_{i}}{\pi }\right)}$$

(4) $$C_{i}=\frac{4\pi A_{i}}{P_{i}^{2}}$$

Image-level pore-structure descriptors were calculated using the PSQI system developed in this study. CV represents pore-size dispersion, Ci represents pore-shape regularity, N represents the number of pores per unit area, UI represents the spatial uniformity of pore number across 4 × 4 subregions, and FL represents the fraction of large-pore defects. Their calculations are given in Equations (5)–(8). CV and FL were treated as cost-type indicators, Ci and UI as benefit-type indicators, and N as a target-type indicator. A single PSQI transform was fitted using 357 physically calibrated development reference images from 98 source groups and was then frozen. The original AHP-Entropy protocol used a non-uniform subjective-importance matrix, with AHP prior weights CV = 0.31069, Ci = 0.11619, N = 0.08748, UI = 0.17496 and FL = 0.31069 (reported CR = 0.01620 in the original method record). Target-type normalization was then performed using Equation (9). After normalization, the integrated score was calculated using Equation (10), where *w*_j_ is the weight and *y*_ij_ is the normalized indicator value. Entropy weights were recalculated from the normalized current development descriptors according to the information-entropy principle [40,41]. Multiplicative normalization yielded the frozen final weights: CV = 0.11767, Ci = 0.03167, N = 0.27144, UI = 0.09924 and FL = 0.47998. *N*_target_ and *N*_delta_ were fixed at 1.59532 and 1.27626 pores mm^−2^, respectively. The frozen PSQI parameters are summarized in Table 2. The same transform was applied unchanged to held-out reference descriptors, held-out FastSAM-DP descriptors and the mixture-image analysis. Because the validation and mix-analysis collections partly share underlying image provenance, the latter is used only for descriptive trend assessment and mix screening and is not treated as an independent validation cohort. Absolute scores from different datasets should not be treated as directly equivalent. The full AHP pairwise-comparison matrix, consistency calculation, indicator normalization and clipping rules, and entropy calculation and fusion rule are provided in the Supplementary Methods.

(5) $$CV=\frac{\sigma_{d}}{d_{\mathrm{av}}}$$

(6) $$N=\frac{n}{A_{s}}$$

(7) $$UI=1-\frac{\sigma_{n}}{n_{\mathrm{av}}}$$

(8) $$FL=\frac{\sum A_{i}(d_{i}>2d_{\mathrm{av}})}{\sum A_{i}}$$

(9) $$y_{N}=\mathrm{clip}\left(1-\frac{\left|N-N_{\mathrm{target}}\right|}{\Delta N},0,1\right),\;\;\;\;\Delta N=0.80N_{\mathrm{target}}$$

(10) $$\mathrm{PSQI}=100\sum w_{j}y_{ij}$$

Reliability was assessed at three levels. At the pixel level, Dice and IoU measured the overlap between predicted masks and manual annotations. At the instance level, precision, recall, Inst-F1 and mAP@0.5 evaluated pore-object recognition. At the parameter level, the CV, Ci, N, UI, FL and PSQI were calculated separately from manual annotations and FastSAM-DP outputs, and consistency was evaluated using the Pearson correlation coefficient, mean absolute error (MAE) and root mean square error (RMSE).

To improve the reproducibility of reference-annotation rules, manual annotation followed predefined constraints. Closed or semi-closed pores with continuous and recognizable pore walls were included. Truncated pores at image edges were retained when their main contours could still be identified, and their area and perimeter were calculated from the visible parts. Regions with severely blurred boundaries, regions indistinguishable from the matrix grayscale and regions that could not be resolved independently were not treated as separate pores. Adjacent pores were annotated separately when a recognizable pore wall existed between them but were treated as one pore instance when the wall disappeared and the region formed a continuous connected area. Because repeated annotation by multiple annotators was not performed, the manual annotations should be understood as reference annotations under a unified rule, rather than as error-free ground truth.

During evaluation, manual annotations were converted into binary masks and instance contours. Instance matching used a greedy rule with IoU ≥ 0.50. Matched predictions, unmatched predicted instances and unmatched manual instances were counted as TP, FP and FN, respectively. Configuration selection used development data only. The 25-group held-out set was evaluated separately and is reported as source-filename-group held-out internal evaluation. A later partition audit mapped each validation source-filename group to its conservative raw-image-field partition and removed held-out groups whose partitions intersected development partitions, defining a post-audit conservative non-overlap sensitivity subset of 14 groups and 56 files. This subset is reported as a sensitivity analysis, not as a pre-specified independent test. Neither result constitutes external validation. All calculations were performed on a Windows 10 workstation equipped with an Intel Core i7-11800H CPU, 16 GB RAM and an NVIDIA GeForce RTX 3060 Laptop GPU. The software environment comprised Python 3.10.20, PyTorch 2.5.1+cu121, CUDA 12.1 and Ultralytics 8.0.120, with FastSAM-s.pt as the FastSAM weight file.

## 3. Results and Discussion

### 3.1. Segmentation Performance and Parameter Selection

**Figure 2:**
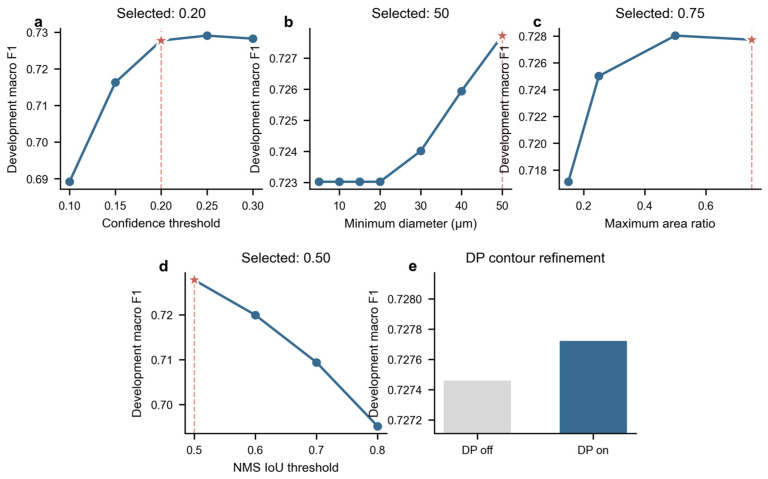
Development-only parameter sensitivity and DP ablation: (**a**) confidence threshold; (**b**) operational minimum diameter; (**c**) maximum prediction-area ratio; (**d**) NMS IoU; (**e**) DP contour refinement. Stars and dashed lines indicate the selected settings.

The inference stability of FastSAM was first examined by varying the confidence and IoU thresholds (Figure 2). The locked workflow was selected using development data only and used confidence = 0.20, NMS IoU = 0.50, a 50 µm operational minimum analysis diameter, a maximum prediction-area ratio of 0.75 and a DP epsilon/perimeter = 0.002. At the audited native scales, 50 µm corresponds to approximately 11.40, 13.65, 17.00, 23.30 and 46.25 pixels at 10×, 12×, 15×, 20× and 40×, respectively. Size-stratified held-out recall remained magnification-dependent, so 50 µm is treated as an analysis cutoff rather than a universal reliable detection limit. All reported segmentation and descriptor results refer to pores within this defined analysis range. Development instance F1 was 0.72772 with DP and 0.72746 without DP, indicating that DP mainly regularized contour geometry rather than materially changing instance recognition.

**Figure 3:**
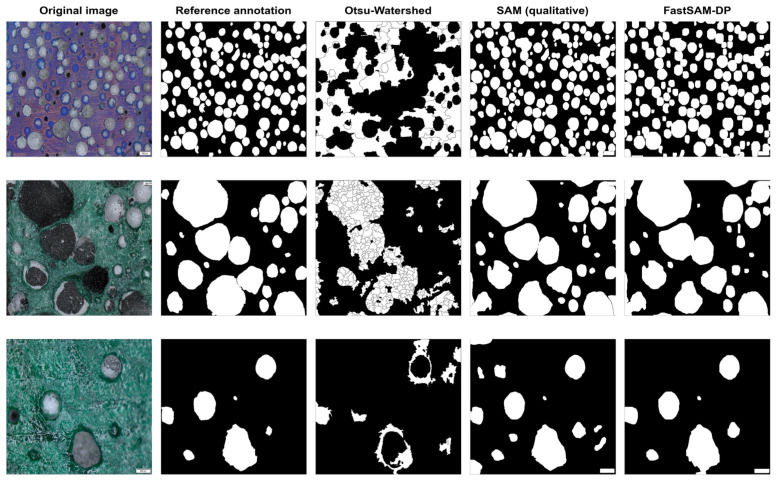
Representative segmentation results. Columns show, from left to right, the original image, reference annotation, Otsu–Watershed, SAM and FastSAM-DP.

The qualitative comparison showed that differences among methods were concentrated in connected-pore separation and small-pore false detection (Figure 3). Otsu–Watershed was sensitive to grayscale contrast and produced large erroneous regions under local shadows and connected-pore walls. SAM suppressed background more effectively, but instance separation between adjacent pores remained unstable. By contrast, FastSAM-DP produced pore boundaries and instance continuity closer to the manual annotations, which is important for subsequent calculations of pore number, perimeter and large-pore fraction.

In Figure 3, each row shows the original image, reference annotation, Otsu–Watershed, SAM and FastSAM-DP results. Quantitative results are reported for the held-out evaluations in Table 3. In the complete 25-group/100-file held-out evaluation, FastSAM-DP achieved precision = 0.726, recall = 0.751, instance F1 = 0.732 (95% CI, 0.696–0.767), matched IoU = 0.857, foreground Dice = 0.867 and AP50 = 0.670; fixed Otsu–Watershed achieved instance F1 = 0.050. In the post-audit 14-group/56-file non-overlap sensitivity subset, FastSAM-DP achieved precision = 0.752, recall = 0.764, instance F1 = 0.752 (95% CI, 0.713–0.791), matched IoU = 0.843, foreground Dice = 0.854 and AP50 = 0.689; fixed Otsu–Watershed achieved instance F1 = 0.051 and foreground Dice = 0.323. Figure 3 is a representative qualitative comparison, not a comprehensive tuned deep-learning benchmark.

The contribution of DP post-processing is therefore not simply higher pixel overlap. DP regularizes and simplifies the polygonal representation of selected contours by reducing pixel-level stair-steps and local burrs. In development, instance F1 was 0.72772 with DP and 0.72746 without DP, so DP should not be interpreted as an instance-splitting or recognition-gain mechanism. Considering accuracy, instance recognition and efficiency together, FastSAM-DP was used for all subsequent parameter extraction.

### 3.2. Automated Validation from Pore Contours to Reference PSQI

After validating segmentation performance, we examined whether automatic masks could support parameter calculation in the PSQI framework defined in this study. To avoid equating mask similarity with material-parameter reliability, CV, Ci, N, UI, FL and the integrated PSQI were calculated separately from manual annotations and FastSAM-DP results, and the two pathways were compared. Manual annotations were used as reference annotations, but this does not mean that every pore boundary was free of subjective judgment. The issue is particularly relevant for truncated edge pores, blurred pore walls and connected-pore regions. Thus, manual annotation in Section 3.2 denotes reference annotation under a unified rule. Its role is to provide a comparable benchmark, rather than absolute ground truth.

**Figure 4:**
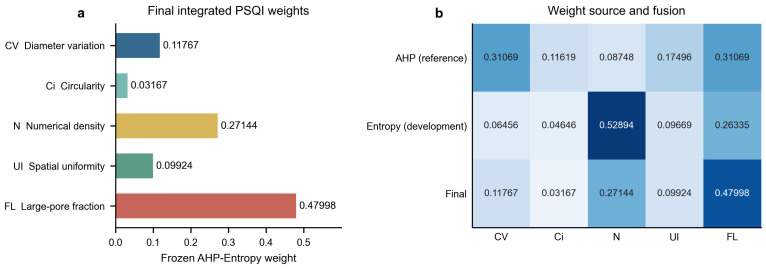
AHP-Entropy weight source and frozen final descriptor weights: (**a**) final integrated weights; (**b**) AHP reference, development entropy and final fused weights.

The AHP-Entropy fused weight structure of the PSQI descriptors is shown in Figure 4. The retained AHP prior weights were 0.31069 for CV, 0.11619 for Ci, 0.08748 for N, 0.17496 for UI and 0.31069 for FL, with CR = 0.01620 reported in the original method record. Recalculated development entropy weights were combined by multiplicative normalization to give frozen final weights of 0.11767 for CV, 0.03167 for Ci, 0.27144 for N, 0.09924 for UI and 0.47998 for FL. *N*_target_ and *N*_delta_ were fixed at 1.59532 and 1.27626 pores mm^−2^, respectively. The model was fitted on development reference annotations and then kept unchanged.

**Figure 5:**
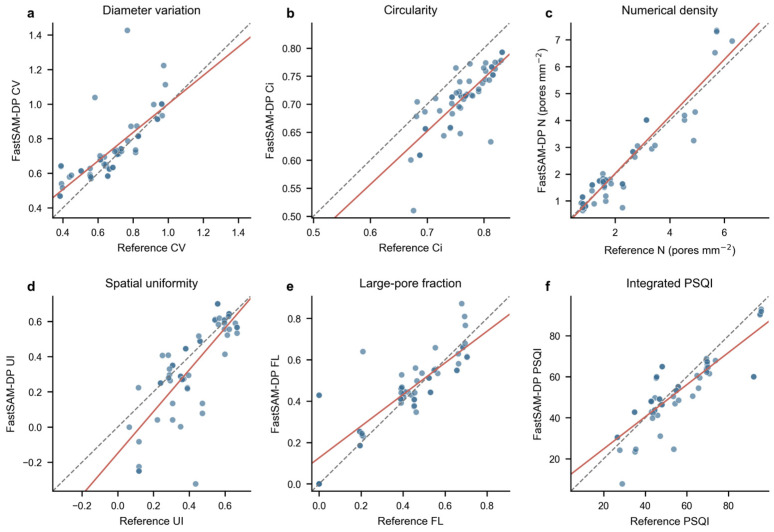
Descriptor agreement between reference annotations and FastSAM-DP: (**a**) CV; (**b**) Ci; (**c**) N; (**d**) UI; (**e**) FL; (**f**) PSQI. Dashed lines indicate identity, red lines indicate linear fits, blue dots represent individual image-level observations.

Agreement between reference annotations and FastSAM-DP extraction for each descriptor is shown in Figure 5. Dashed lines indicate ideal agreement, and red lines indicate linear fits. In the conservative 14-group non-overlap sensitivity subset, Pearson correlations were 0.751 for CV, 0.785 for Ci, 0.944 for N, 0.775 for UI and 0.837 for FL. N and UI mainly depend on pore-instance number and spatial distribution, whereas CV, Ci and FL are more sensitive to contour boundaries, a small number of large pores and connected-pore separation. Subjective decisions in the reference protocol, such as whether to retain edge-truncated or poorly separated pores, may partly contribute to these differences. Because no second-annotator study was available, this contribution cannot be quantified or separated from algorithm error. Because multi-annotator consistency was not evaluated, these correlations should be interpreted as reproducibility relative to the current reference annotations.

**Figure 6:**
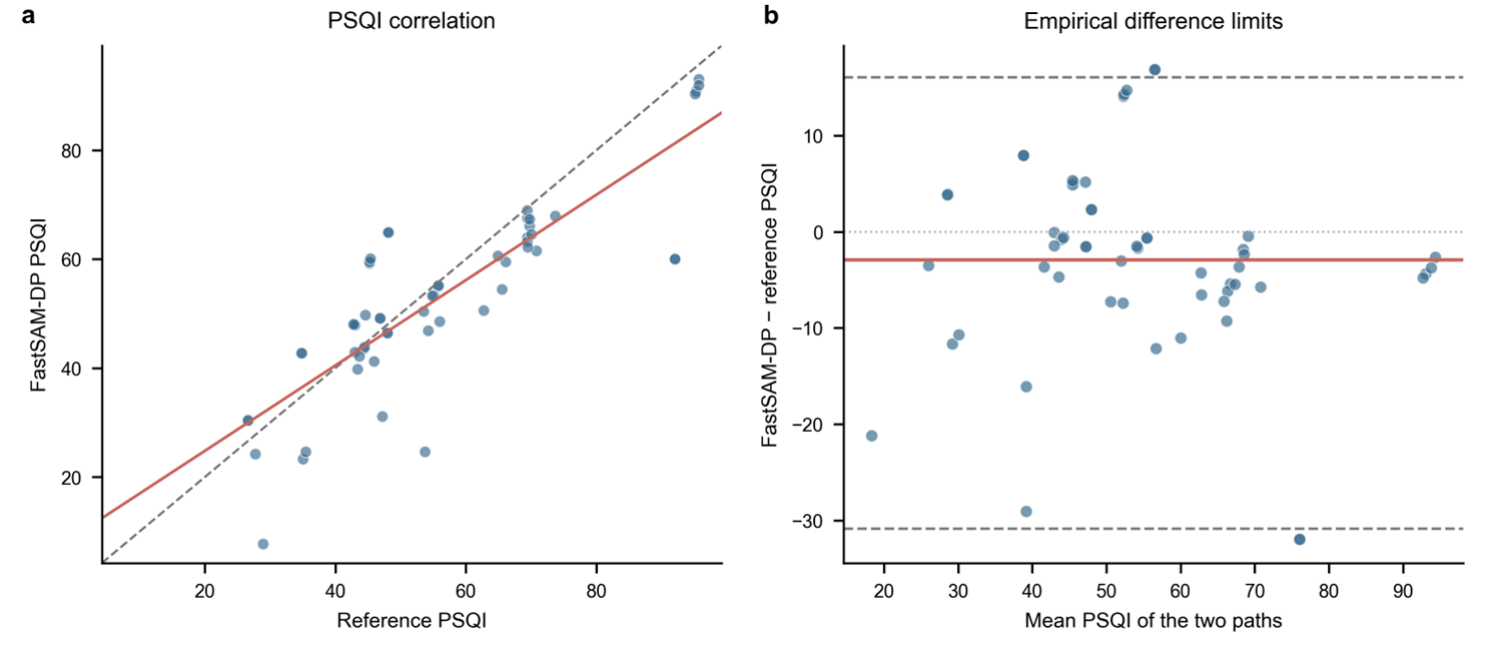
PSQI agreement between reference annotations and FastSAM-DP: (**a**) reference versus FastSAM-DP PSQI; (**b**) difference versus mean PSQI. Blue dots represent individual image-level observations; dashed lines indicate identity, red lines indicate linear fits.

Integrated PSQI consistency is shown in Figure 6. In the complete 25-group/100-file held-out evaluation, reference and FastSAM-DP PSQI values had Pearson r = 0.828 (95% CI, 0.665–0.917), Spearman ρ = 0.837, MAE = 7.94 and RMSE = 11.10. In the post-audit 14-group/56-file non-overlap sensitivity subset, Pearson r = 0.838 (95% CI, 0.585–0.942), Spearman ρ = 0.845, MAE = 7.41 and RMSE = 10.50. The mean difference was −2.92 points, with empirical 2.5th and 97.5th percentile limits of −30.86 and 16.06 points. This consistency was lower than that of N and UI because the PSQI combines multiple boundary-sensitive and large-pore-sensitive descriptors into a single score. Reference-annotation subjectivity may account for part of the observed difference, but algorithm error and reference uncertainty cannot be separated without repeated independent annotation. The PSQI is therefore better suited to batch screening and trend comparison than to replacing manual review or fine interpretation of single images. In the absence of external measured links to compressive strength, dry density, thermal conductivity or durability, the PSQI is used here only as an image-scale integrated descriptor. Future validation should use prospectively collected, image-matched specimens with batch tracking, standardized imaging and paired mechanical testing to evaluate whether the PSQI is associated with compressive strength independently of density.

Overall, the automatic workflow reproduced pore-number density and spatial-uniformity descriptors more consistently than boundary and large-pore-sensitive descriptors in the present archive. For the large-pore fraction, pore-size dispersion and integrated PSQI, it is more appropriate for trend judgment and same-magnification mix screening than for full replacement of manual annotation. A future multi-annotator study is needed to distinguish algorithm error from reference-annotation variability. If higher consistency in integrated scores is required, improving large-pore recognition, connected-pore separation and boundary smoothing should be prioritized.

### 3.3. Scale Sensitivity of Pore-Structure Indicators Under Multiscale Observation

After segmentation and parameter-consistency validation, FastSAM-DP was applied to multiscale analysis of the HP, SF and WS mix series. The analysis contained 130 mixture micrographs grouped into 107 conservative image-field partitions: 39 files/30 partitions at 15×, 45/37 at 20× and 46/40 at 40×. Rather than directly ranking individual mixes, this section uses 15×, 20× and 40× as the main explanatory conditions and examines observation-scale sensitivity in the CV, Ci, N, UI, FL and PSQI. This treatment helps avoid misinterpreting scale effects as intrinsic material differences. The archived records were insufficient to establish exact spatial correspondence across magnifications. Accordingly, the magnification strata were analyzed as independent descriptive observations within the same mix systems rather than as a single-factor causal experiment on magnification.

**Figure 7:**
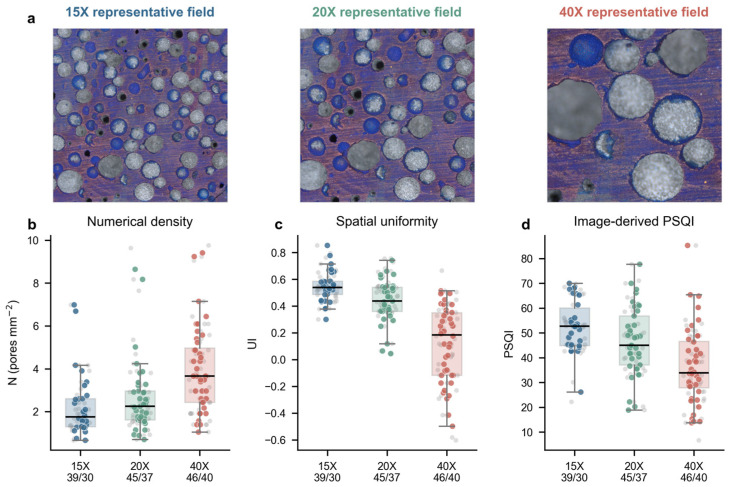
Representative archive fields and descriptor distributions across observation scales: (**a**) fields at 15×, 20× and 40×; (**b**) N; (**c**) UI; (**d**) PSQI. Grey points are image-level records; colored points and boxes are partition-level summaries.

The overall response of pore-structure indicators under multiscale observation is shown in Figure 7. Panel (a) shows representative archive fields at 15×, 20× and 40×, whereas panels (b)–(d) show independent image-level observations together with partition-level summaries of N, UI and PSQI. The archive comprised 130 image records and 107 partition-level summaries (15×: 39/30 files/partitions; 20×: 45/37; 40×: 46/40). No lines or 40×-minus-15× differences are used. The three magnification strata were analyzed independently, so the distributions describe combined observation-scale and sampled-region variation rather than causal magnification effects.

**Figure 8:**
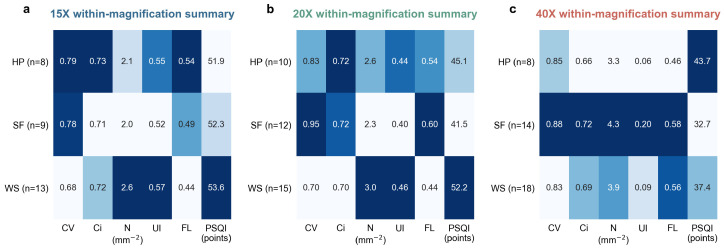
HP, SF and WS descriptor profiles within each magnification: (**a**) 15×; (**b**) 20×; (**c**) 40×. Cell values are partition-level means, and n is the number of partitions.

Figure 8 summarizes HP, SF and WS descriptor profiles separately within 15×, 20× and 40× magnifications. Each panel uses its own descriptor-wise color normalization, and the displayed values are means of partition-level summaries; n denotes the number of partitions. This arrangement supports within-magnification comparison of the three series without using a common color scale to rank results across magnifications.

**Figure 9:**
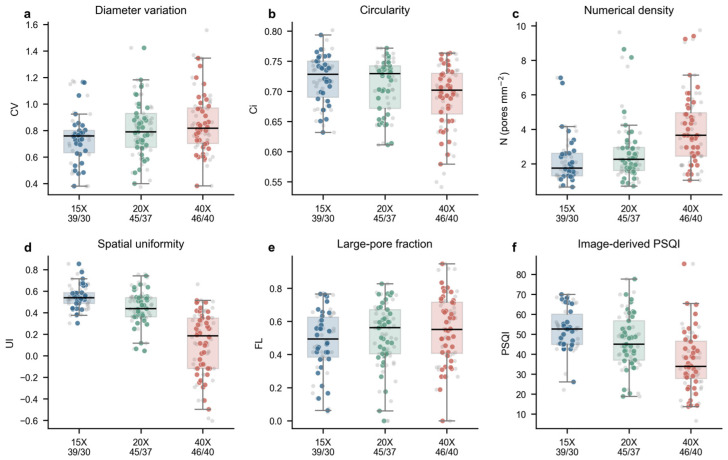
Descriptor distributions at 15×, 20× and 40×: (**a**) CV; (**b**) Ci; (**c**) N; (**d**) UI; (**e**) FL; (**f**) PSQI. Grey points are image-level records; colored points and boxes are partition-level summaries.

Figure 9 replaces the former 40×-minus-15× analysis with independent distributions of the CV, Ci, N, UI, FL and PSQI at each magnification. The panels retain all 130 image observations and overlay the 107 partition-level summaries. They describe sampled range and dispersion, not paired change or an intrinsic material response to magnification.

**Figure 10:**
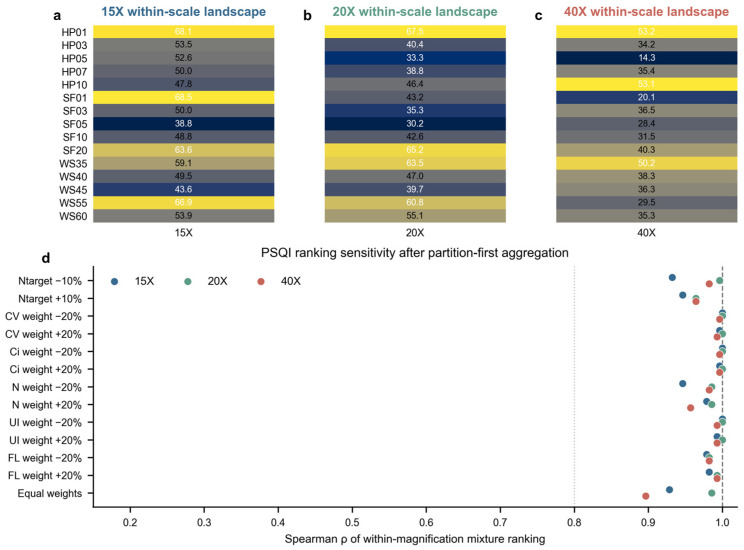
Within-magnification PSQI landscapes and sensitivity analysis: (**a**) 15×; (**b**) 20×; (**c**) 40×; (**d**) rank stability under parameter and weight perturbations.

Figure 10 reports separate within-magnification PSQI landscapes for the 15 nominal series × dose levels. Panels (a)–(c) show partition-first mean PSQI at 15×, 20× and 40×, respectively; colors are normalized within each magnification, and printed numbers are PSQI values. Panel (d) shows the Spearman ρ between the baseline and perturbed within-magnification rankings. After partition-first aggregation, *N*_target_ ±10% gave ρ = 0.932–0.996, single final-weight ±20% perturbations gave ρ = 0.946–1.000, and equal weighting as a different framework gave ρ = 0.896–0.986. These results indicate local stability under local perturbations but dependence on the overall weighting framework; no cross-magnification ranking was performed.

In summary, the independently sampled 15×, 20× and 40× archives showed different descriptor distributions. These differences combine observation scale and sampled-region variability and should not be interpreted as paired material changes. FastSAM-DP and the PSQI are therefore more appropriate for mix screening within the same magnification. If the objective is to compare mix differences, magnification, field-of-view selection rules and image numbers should be fixed as far as possible.

## 4. Conclusions

This study developed an analytical workflow for automatic pore-structure characterization of foamed-concrete micrographs. The workflow integrates FastSAM candidate segmentation, DP contour correction, extraction of CV/Ci/N/UI/FL descriptors and data-driven integrated PSQI assessment. It supports batch pore-structure screening and same-magnification comparison within the present internal dataset. Performance was assessed by grouped held-out internal evaluation with an additional post-audit non-overlap sensitivity analysis. PSQI remains an image-derived composite descriptor rather than a validated material-performance index.

(1) A FastSAM-DP workflow was developed for automatic pore segmentation and parameter extraction in foamed concrete. It enables continuous conversion from micrographs and pore instance masks to pore-level geometric information, CV/Ci/N/UI/FL descriptors and integrated PSQI. The workflow reduces reliance on operator experience in manual outlining and empirical threshold segmentation, providing a reproducible technical route for batch pore-structure analysis of foamed concrete.
(2) In the complete 25-group/100-file held-out evaluation, FastSAM-DP achieved instance F1 = 0.732 (95% CI, 0.696–0.767), matched IoU = 0.857, foreground Dice = 0.867 and AP50 = 0.670, whereas fixed Otsu–Watershed achieved instance F1 = 0.050. In the post-audit 14-group/56-file non-overlap sensitivity subset, FastSAM-DP achieved instance F1 = 0.752 (95% CI, 0.713–0.791), matched IoU = 0.843, foreground Dice = 0.854 and AP50 = 0.689, whereas fixed Otsu–Watershed achieved instance F1 = 0.051. DP regularized and simplified contour geometry rather than materially increasing instance recognition. Independent external data are still needed to verify applicability across imaging devices and sample batches.
(3) In the complete 25-group/100-file held-out evaluation, PSQI agreement with the current reference protocol was r = 0.828 (95% CI, 0.665–0.917), with MAE = 7.94 and RMSE = 11.10. In the post-audit 14-group/56-file non-overlap sensitivity subset, r = 0.838 (95% CI, 0.585–0.942), with MAE = 7.41 and RMSE = 10.50. Reference-annotation subjectivity may explain part of the difference, but its contribution cannot be separated from algorithm error without a second annotator. The automatic workflow is therefore intended for batch screening and trend assessment rather than fine interpretation of individual images.
(4) The independently sampled 15×, 20× and 40× strata comprised 130 images and 107 conservative image-field partitions and showed different descriptor distributions. Because the magnification strata were analyzed as independent descriptive observations, the differences combine observation-scale and local-region variation. Cross-magnification results should not be used to rank material quality, and mix comparisons should preferably be conducted at the same magnification.

## Figures and Tables

**Table 1:** HP/SF/WS sample grouping and numbers of valid images.

Mixture Level	15× Files/Partitions	20× Files/Partitions	40× Files/Partitions	Total Files
HP01	2/1	2/1	2/2	6
HP03	1/1	2/1	2/1	5
HP05	2/1	1/1	1/1	4
HP07	2/1	2/2	2/1	6
HP10	5/4	5/5	4/3	14
SF01	1/1	2/2	1/1	4
SF03	2/2	3/3	3/3	8
SF05	4/2	3/3	4/4	11
SF10	4/2	4/2	4/3	12
SF20	2/2	3/2	3/3	8
WS35	2/2	2/2	3/3	7
WS40	2/2	3/2	3/2	8
WS45	3/3	5/4	5/5	13
WS55	3/2	3/3	4/3	10
WS60	4/4	5/4	5/5	14
Total	39/30	45/37	46/40	130/107

**Table 2:** Frozen PSQI parameters fitted from development reference annotations.

Descriptor	Polarity	Frozen Bound	AHP Prior	Final Weight
CV	Cost	0.31891–1.60421	0.31069	0.11767
Ci	Benefit	0.58931–0.84426	0.11619	0.03167
N	Target	0.44319–11.64391 pores mm^−2^	0.08748	0.27144
UI	Benefit	−0.59861–0.80753	0.17496	0.09924
FL	Cost	0–0.87949	0.31069	0.47998

**Table 3:** Source-group macro-segmentation results for the complete held-out evaluation and the post-audit non-overlap sensitivity subset.

Evaluation Subset	Method	Precision	Recall	Instance F1	Matched IoU	Foreground Dice	Foreground IoU	AP50
Internal held-out set (25 groups, 100 files)	FastSAM-DP	0.726	0.751	0.732	0.857	0.867	0.768	0.670
	Otsu–Watershed	0.047	0.094	0.050	0.698	0.337	0.222	-
Post-audit non-overlap sensitivity subset (14 groups, 56 files)	FastSAM-DP	0.752	0.764	0.752	0.843	0.854	0.748	0.689
	Otsu–Watershed	0.044	0.094	0.051	0.698	0.323	0.209	-

## Data Availability

Supplementary Methods and Supplementary Data accompanying this submission provide the fixed configuration, annotation protocol, image-to-group and image-field-partition mapping, development/held-out and post-audit assignments, image-level held-out outputs, mix-analysis descriptors and size-stratified recall results. The underlying raw image archive and analysis code are available from the corresponding authors upon reasonable request, subject to applicable repository and data-sharing restrictions.

## Supplementary Materials

The following supporting information can be downloaded at https://www.mdpi.com/article/10.3390/ma19153215/s1, S1. Operational definitions and data grouping. S2. Validation–mix provenance audit. S3. Fixed analysis configuration and reference-annotation protocol. S4. Operational minimum diameter and size-stratified recall. S5. Douglas–Peucker processing. S6. PSQI reproducibility. S7. Accompanying data tables.

## Abbreviations

The following abbreviations are used in this manuscript:

FastSAM	Fast Segment Anything Model
DP	Douglas–Peucker
PSQI	Image-derived composite pore-structure descriptor
CV	Coefficient of variation
Ci	Circularity index
N	Pore-number density
UI	Uniformity index
FL	Large-pore fraction

## References

[1] Hou L., Li J., Lu Z., Niu Y. (2021). Influence of Foaming Agent on Cement and Foam Concrete. Constr. Build. Mater..

[2] Ramamurthy K., Kunhanandan Nambiar E.K., Indu Siva Ranjani G. (2009). A Classification of Studies on Properties of Foam Concrete. Cem. Concr. Compos..

[3] Amran Y.H.M., Farzadnia N., Abang Ali A.A. (2015). Properties and Applications of Foamed Concrete; a Review. Constr. Build. Mater..

[4] Xiong L., Fan B., Wan Z., Zhang Z., Zhang Y., Shi P. (2021). Study on the Mechanical Properties of Fly-Ash-Based Light-Weighted Porous Geopolymer and Its Utilization in Roof-Adaptive End Filling Technology. Molecules.

[5] Xu J., Zhao D., Wang S., Chen X., Wu X., Han Z., Liu Y. (2025). Quantitative Analysis of Pore Structure’s Impact on Early Mechanical Properties and Durability of Foam Concrete: Macroscopic and Mesoscopic Insights. Constr. Build. Mater..

[6] Nambiar E.K.K., Ramamurthy K. (2007). Air-void Characterisation of Foam Concrete. Cem. Concr. Res..

[7] Hilal A.A., Thom N.H., Dawson A.R. (2015). On Entrained Pore Size Distribution of Foamed Concrete. Constr. Build. Mater..

[8] Fan D., Zhang C., Lu J.-X., Peng L., Yu R., Poon C.S. (2025). Rheology Dependent Pore Structure Optimization of High-Performance Foam Concrete. Cem. Concr. Res..

[9] Zhang C., Fan D., Lu J.-X., Pang C., Poon C.S. (2024). Ultra-Stable Foam Enabled by Nano Silica Engineering for Foam Concrete Improvement. Cem. Concr. Compos..

[10] Lv X.-S., Cao W.-X., Yio M., Ji W.-Y., Lu J.-X., She W., Poon C.S. (2024). High-Performance Lightweight Foam Concrete Enabled by Compositing Ultra-Stable Hydrophobic Aqueous Foam. Cem. Concr. Compos..

[11] She W., Zheng Z., Zhang Q., Zuo W., Yang J., Zhang Y., Zheng L., Hong J., Miao C. (2020). Predesigning Matrix-Directed Super-Hydrophobization and Hierarchical Strengthening of Cement Foam. Cem. Concr. Res..

[12] Zhang S., Cao K., Wang C., Wang X., Deng G., Wei P. (2020). Influence of the Porosity and Pore Size on the Compressive and Splitting Strengths of Cellular Concrete with Millimeter-Size Pores. Constr. Build. Mater..

[13] Kearsley E.P., Wainwright P.J. (2001). Porosity and Permeability of Foamed Concrete. Cem. Concr. Res..

[14] Kearsley E.P., Wainwright P.J. (2002). The Effect of Porosity on the Strength of Foamed Concrete. Cem. Concr. Res..

[15] Otsu N. (1979). A Threshold Selection Method from Gray-Level Histograms. IEEE Trans. Syst. Man Cybern..

[16] Ronneberger O., Fischer P., Brox T. U-Net: Convolutional Networks for Biomedical Image Segmentation. Proceedings of the Medical Image Computing and Computer-Assisted Intervention—MICCAI 2015.

[17] He K., Gkioxari G., Dollár P., Girshick R. Mask R-CNN. Proceedings of the 2017 IEEE International Conference on Computer Vision (ICCV).

[18] Qian H., Li Y., Yang J., Xie L., Tan K.H. (2023). Segmentation and Analysis of Cement Particles in Cement Paste with Deep Learning. Cem. Concr. Compos..

[19] Zhang H., Zhang R., Sun D., Yu F., Gao Z., Sun S., Zheng Z. (2022). Analyzing the Pore Structure of Pervious Concrete Based on the Deep Learning Framework of Mask R-CNN. Constr. Build. Mater..

[20] Bangaru S.S., Wang C., Zhou X., Hassan M. (2022). Scanning Electron Microscopy (SEM) Image Segmentation for Microstructure Analysis of Concrete Using U-Net Convolutional Neural Network. Autom. Constr..

[21] Hilloulin B., Bekrine I., Schmitt E., Loukili A. (2022). Modular Deep Learning Segmentation Algorithm for Concrete Microscopic Images. Constr. Build. Mater..

[22] Kirillov A., Mintun E., Ravi N., Mao H., Rolland C., Gustafson L., Xiao T., Whitehead S., Berg A.C., Lo W.-Y. Segment Anything. Proceedings of the 2023 IEEE/CVF International Conference on Computer Vision (ICCV).

[23] Zhao X., Ding W., An Y., Du Y., Yu T., Li M., Tang M., Wang J. (2023). Fast Segment Anything. arXiv.

[24] Papia E.-M., Kondi A., Constantoudis V. (2025). Machine Learning Applications in SEM-Based Pore Analysis: A Review. Microporous Mesoporous Mater..

[25] Ullah M., Plevris V., Mir J., Ahmad A. (2026). Image-Based Prediction of Concrete Mechanical Properties through CNN Segmentation and ANN Modeling. Results Eng..

[26] Ma Y., Qian H., Zou D., Zhou A., Liu T., Li Y. (2025). Resolution Enhancement of Cementitious Microstructure Images and Phases Quantification Using Deep Learning. Constr. Build. Mater..

[27] Nguyen T., Kashani A., Ngo T., Bordas S. (2019). Deep Neural Network with High-Order Neuron for the Prediction of Foamed Concrete Strength. Comput.-Aided Civ. Infrastruct. Eng..

[28] Wang X., Xiao Y., Li W., Wang M., Zhou Y., Chen Y., Li Z. (2024). Kriging-Based Surrogate Data-Enriching Artificial Neural Network Prediction of Strength and Permeability of Permeable Cement-Stabilized Base. Nat. Commun..

[29] Kakasor Ismael Jaf D., Ismael Abdulrahman P., Salih Mohammed A., Kurda R., Qaidi S.M.A., Asteris P.G. (2023). Machine Learning Techniques and Multi-Scale Models to Evaluate the Impact of Silicon Dioxide (SiO_2_) and Calcium Oxide (CaO) in Fly Ash on the Compressive Strength of Green Concrete. Constr. Build. Mater..

[30] Song H., Ahmad A., Farooq F., Ostrowski K.A., Maślak M., Czarnecki S., Aslam F. (2021). Predicting the Compressive Strength of Concrete with Fly Ash Admixture Using Machine Learning Algorithms. Constr. Build. Mater..

[31] Wawrzeńczyk J., Kowalczyk H. (2025). Estimation of the Spacing Factor Based on Air Pore Distribution Parameters in Air-Entrained Concrete. Materials.

[32] Dhasindrakrishna K., Pasupathy K., Ramakrishnan S., Sanjayan J. (2021). Progress, Current Thinking and Challenges in Geopolymer Foam Concrete Technology. Cem. Concr. Compos..

[33] Dhasindrakrishna K., Ramakrishnan S., Pasupathy K., Sanjayan J. (2021). Collapse of Fresh Foam Concrete: Mechanisms and Influencing Parameters. Cem. Concr. Compos..

[34] Xiong L., Maltseva O., Chen B., Fernández-Jiménez A., Wan Z., Palomo A. (2025). Modelling the Hydration of Ultra-Low Clinker Cements: The Impact of Sodium Salts Additives. Constr. Build. Mater..

[35] Xiong L., Maltseva O., Fernandez-Jimenez A., Wan Z., Palomo A. (2025). Transforming Landfilled Coal Ash in Ultralow Clinker Binders. J. Am. Ceram. Soc..

[36] Falliano D., De Domenico D., Ricciardi G., Gugliandolo E. (2018). Experimental Investigation on the Compressive Strength of Foamed Concrete: Effect of Curing Conditions, Cement Type, Foaming Agent and Dry Density. Constr. Build. Mater..

[37] Kearsley E.P., Wainwright P.J. (2001). The Effect of High Fly Ash Content on the Compressive Strength of Foamed Concrete. Cem. Concr. Res..

[38] Nambiar E.K.K., Ramamurthy K. (2006). Influence of Filler Type on the Properties of Foam Concrete. Cem. Concr. Compos..

[39] Douglas D.H., Peucker T.K. (1973). Algorithms for the Reduction of the Number of Points Required to Represent a Digitized Line or Its Caricature. Cartographica.

[40] Shannon C.E. (1948). A Mathematical Theory of Communication. Bell Syst. Tech. J..

[41] Saaty T.L. (2008). Decision Making with the Analytic Hierarchy Process. Int. J. Serv. Sci..

