# Supplementary material for: FastSAM-Based Automated Segmentation and Data Extraction for Pore Structures of Foamed Concrete
Source: Materials (Basel). 2026 Jul 28;19(15):3215. doi: 10.3390/ma19153215 (PMC13467136; doi:10.3390/ma19153215)
Supplement: Supplementary file 1 [file materials-19-03215-s001.zip › materials-4438737-supplementary.pdf]

Supplementary Methods

FastSAM-DP pore segmentation and pore-structure assessment: reproducibility additions for the second-round revision

S1. Operational definitions and data grouping

A source-filename group comprises all 640 × 640 validation exports sharing the filename before the `.rf.<hash>` suffix. A conservative image-field partition is a connected set of raw-image records judged identical, spatially overlapping or ambiguous on the basis of filename provenance and geometric audit evidence. Post-audit overlap screening mapped validation groups to raw-image partitions and removed held-out groups whose partitions intersected development partitions. The full mapping is supplied in Supplementary Data.

S2. Validation–mix provenance audit

The validation collection contained 473 JPG exports in 125 source-filename groups. The retained mix-analysis collection contained 130 BMP images in 107 conservative partitions. No cross-collection file pair was identical by SHA-256. Source-name tracing nevertheless linked 108 validation groups (432 exports) to 108 of the 130 mix-analysis images. The two collections therefore differ in representation and purpose but partly share underlying image provenance. Mix analysis is descriptive and is not an independent validation cohort.

S3. Fixed analysis configuration and reference-annotation protocol

Item	Fixed rule
FastSAM	FastSAM-s.pt; confidence ≥0.20; NMS IoU 0.50
Operational diameter	Equivalent diameter ≥50 µm for both reference and predicted instances
Prediction-only filter	Image-area ratio ≤0.75
Matching	Greedy one-to-one instance matching at IoU ≥0.50
Douglas–Peucker	Tolerance = 0.002 × native perimeter; contour simplification only
Reference annotation	Continuous recognizable walls included; visible portions of identifiable edge pores retained; unresolvable regions excluded; adjacent pores separated only when a recognizable wall exists

S4. Operational minimum diameter and size-stratified recall

The 50 µm cutoff was applied symmetrically to reference and predicted instances before matching, segmentation metrics and descriptor calculation. Confidence and maximum-area filters applied only to predictions. Reported performance therefore pertains to pores with equivalent diameter ≥50 µm. Recall remains magnification- and size-dependent, and the cutoff is not a universal detection limit.

Subset	Mag.	Diameter (µm)	Matched/eligible	Recall
complete 25-group held-out	15X	≥250	1621/1834	0.884
complete 25-group held-out	15X	150–<250	383/504	0.760
complete 25-group held-out	15X	100–<150	72/219	0.329

Subset	Mag.	Diameter (µm)	Matched/eligible	Recall
complete 25-group held-out	15X	75–<100	6/43	0.140
complete 25-group held-out	40X	≥250	125/146	0.856
complete 25-group held-out	40X	150–<250	35/52	0.673
complete 25-group held-out	40X	100–<150	23/41	0.561
complete 25-group held-out	10X	≥250	653/721	0.906
complete 25-group held-out	10X	150–<250	60/91	0.659
complete 25-group held-out	10X	100–<150	0/1	0.000
complete 25-group held-out	40X	50–<75	0/5	0.000
complete 25-group held-out	20X	≥250	738/976	0.756
complete 25-group held-out	20X	150–<250	412/520	0.792
complete 25-group held-out	20X	100–<150	193/296	0.652
complete 25-group held-out	20X	75–<100	23/40	0.575
complete 25-group held-out	40X	75–<100	0/8	0.000
complete 25-group held-out	15X	50–<75	0/23	0.000
complete 25-group held-out	20X	50–<75	5/10	0.500
post-audit 14-group subset	40X	≥250	97/112	0.866
post-audit 14-group subset	40X	150–<250	35/42	0.833
post-audit 14-group subset	40X	100–<150	23/41	0.561
post-audit 14-group subset	10X	≥250	653/721	0.906
post-audit 14-group subset	10X	150–<250	60/91	0.659
post-audit 14-group subset	10X	100–<150	0/1	0.000
post-audit 14-group subset	20X	≥250	454/625	0.726
post-audit 14-group subset	20X	150–<250	226/274	0.825
post-audit 14-group subset	20X	100–<150	144/199	0.724
post-audit 14-group subset	20X	75–<100	21/34	0.618
post-audit 14-group subset	40X	75–<100	0/8	0.000
post-audit 14-group subset	40X	50–<75	0/4	0.000
post-audit 14-group subset	20X	50–<75	4/5	0.800
post-audit 14-group subset	15X	≥250	428/504	0.849
post-audit 14-group subset	15X	150–<250	115/144	0.799
post-audit 14-group subset	15X	75–<100	4/11	0.364
post-audit 14-group subset	15X	100–<150	28/42	0.667
post-audit 14-group subset	15X	50–<75	0/12	0.000

S5. Douglas–Peucker processing

DP did not split or merge instances. Development instance F1 was 0.72772 with DP and 0.72746 without DP. Its stated role is therefore limited to regularizing and simplifying selected polygonal contours by reducing pixel-level stair-steps and local burrs, not improving recognition.

S6. PSQI reproducibility

Indicators are ordered as CV, Ci, N, UI and FL. The reciprocal AHP matrix was

	CV	Ci	N	UI	FL
CV	1	3	3	2	1
Ci	1/3	1	2	1/2	1/3
N	1/3	1/2	1	1/2	1/3
UI	1/2	2	2	1	1/2
FL	1	3	3	2	1

AHP weights were obtained by column normalization followed by row averaging: (0.31069, 0.11619, 0.08748, 0.17496, 0.31069). The weighted-sum consistency calculation gave λmax = 5.07258, CI = (λmax − 5)/4 = 0.01815, RI5 = 1.12 and CR = CI/RI = 0.01620.

For benefit indicators, y = (x − L)/(U − L); for cost indicators, y = (U − x)/(U − L); and for the target indicator N, y = 1 − |x − Ntarget|/Ndelta. Every y was clipped to [0,1]. If U = L, y was set to 0.5. Entropy used 0 ln 0 = 0. Entropy weights were calculated from normalized development reference descriptors, and final weights were obtained by normalizing the elementwise product wAHP × wentropy. Ntarget = 1.59532 and Ndelta = 1.27626 pores mm^{-2}. The frozen final weights were CV 0.11767, Ci 0.03167, N 0.27144, UI 0.09924 and FL 0.47998.

S7. Accompanying data tables

Supplementary Data contains the fixed configuration, annotation protocol, 473-image validation mapping, development/held-out and post-audit assignments, 130-image mix mapping, held-out image-level segmentation and descriptor outputs, size-stratified recall, the AHP matrix, and PSQI bounds and weights.
